# Balance between MKK6 and MKK3 Mediates p38 MAPK Associated Resistance to Cisplatin in NSCLC

**DOI:** 10.1371/journal.pone.0028406

**Published:** 2011-12-02

**Authors:** Eva M. Galan-Moya, Miguel A. de la Cruz-Morcillo, Maria Llanos Valero, Juan L. Callejas-Valera, Pedro Melgar-Rojas, Javier Hernadez Losa, Mayte Salcedo, Antonio Fernández-Aramburo, Santiago Ramon y. Cajal, Ricardo Sánchez-Prieto

**Affiliations:** 1 Laboratorio de Oncología Molecular, Centro Regional de Investigaciones Biomédicas, PCYTA/ UCLM, Albacete, Spain; 2 Pathology Department, Fundació Institut de Recerca Hospital Vall d'Hebron, Barcelona, Spain; 3 Servicio de Oncología CHUA, Albacete, Spain; Roswell Park Cancer Institute, United States of America

## Abstract

The p38 MAPK signaling pathway has been proposed as a critical mediator of the therapeutic effect of several antitumor agents, including cisplatin. Here, we found that sensitivity to cisplatin, in a system of 7 non-small cell lung carcinoma derived cell lines, correlated with high levels of MKK6 and marked activation of p38 MAPK. However, knockdown of MKK6 modified neither the response to cisplatin nor the activation of p38 MAPK. Deeper studies showed that resistant cell lines also displayed higher basal levels of MKK3. Interestingly, MKK3 knockdown significantly decreased p38 phosphorylation upon cisplatin exposure and consequently reduced the response to the drug. Indeed, cisplatin poorly activated MKK3 in resistant cells, while in sensitive cell lines MKK3 showed the opposite pattern in response to the drug. Our data also demonstrate that the low levels of MKK6 expressed in resistant cell lines are the consequence of high basal activity of p38 MAPK mediated by the elevated levels of MKK3. This finding supports the existence of a regulatory mechanism between both MAPK kinases through their MAPK. Furthermore, our results were also mirrored in head and neck carcinoma derived cell lines, suggesting our observations boast a potential universal characteristic in cancer resistance of cisplatin. Altogether, our work provides evidence that MKK3 is the major determinant of p38 MAPK activation in response to cisplatin and, hence, the resistance associated with this MAPK. Therefore, these data suggest that the balance between both MKK3 and MKK6 could be a novel mechanism which explains the cellular response to cisplatin.

## Introduction

Cisplatin (cDDP) and its derivates are between the most used drugs in cancer therapy, although the frequent development of resistance to the drug is one of its major limitations [1]. Among the several pathologies currently treated with cDDP, non-small cell lung carcinoma (NSCLC), a subtype of lung cancer, is one of the most challenging, due to the high ratio of refractory patients to the current therapy (for a review see [2]). Nonetheless, new approaches have been proposed to overcome cDDP resistance, such as the use of glytazonas or copper chelators [3]–[5], that that are aimed to improve cDDP based therapy, allowing more selective use of the drug and avoiding some of the side effects. The main target of cDDP is the DNA molecule, where the drug induces several types of damages -mainly inter and intra catenaries crosslinks- which prevent cell growth (for a review [6]). Resistance to cDDP has been suggested to involve the dysfunction of several genes which are major determinants of DNA repair mechanism, such as p53, ERCC1 or BRCA1, and indeed some of these have already been proposed as markers for patient outcome in cDDP based therapy [7]–[9]. In addition to these molecules, several signaling pathways have also been implicated in cDDP resistance including the MAPK (Mitogen Activated Protein Kinase) family [10]. In this regard, one of the member of the MAPK family, p38 MAPK, has been demonstrated to be a key player in the cellular response to cDDP, and therefore in cancer therapy [11]. In fact, previous reports showed how this signaling pathway is implicated in cDDP resistance [12] and how it is connected to molecules critical in tumor growth and the response to cDDP, such as p53 or c-Abl [13], [14] and more recently with the epidermal growth factor receptor (EGFR), which has indeed become a new target in lung cancer therapy [15], [16]. Furthermore, it has been demonstrated that MKP1, a phosphatase linked to several MAPK, including p38 MAPK, is implicated in the cellular response to cDDP in a NSCLC derived cell line [17]. Interestingly, both natural activators of p38 MAPK, the MAPK kinases (MAPKK) MKK6 and MKK3, show a very high structure similarity and are in fact activated both in *vivo* and *in vitro* by the same stimuli, including cDDP, TNF and UV [12], [18]. However, one of the few differences between both MAPKKs, is that MKK6 is regulated at the transcriptional level by p38 MAPK activity [19], suggesting the existence of a regulatory mechanism between the MAPK and this particular MAPKK. The p38 MAPK pathway has been connected with cancer development (for a review [20]) and in the particular case of lung cancer seems to be a critical protein in the control of stem and progenitor cells [21]. In fact, the existence of hyperactivation of this MAPK in lung cancer samples has been reported [22], although so far no definitive link has been established between p38 MAPK and patient outcome [23]. Therefore, the identification of new markers within the pathway could be extremely useful to validate the role of p38 MAPK signaling network as a biomarker in lung cancer and its therapy.

Here, we present evidence that MKK3, but not MKK6, is the critical player in p38 MAPK signaling-associated cDDP resistance in NSCLC derived cell lines. Our data also demonstrate that the interplay between both MAPKKs is crucial for p38 MAPK activation in response to cDDP and proposes that the imbalance between MKK6/MKK3 could be a potential biomarker in NSCLC.

## Materials and Methods

### Cell lines

Non-small lung cancer cells (H23, Hop62, H157, H226, H460, H661 and H1299) and Head and neck squamous cell carcinoma cells (HN12, HN19, HN26 and HN30) were maintained in DMEM (Invitrogen, Carlsbad, CA) supplemented with 10% FCS plus antibiotics (Biowhittaker, Verviers, Belgium). Cells were maintained in 5% CO_2_ and 37°C. H460 and H1299 were purchased from ATCC (LGC promochem, Barcelona, Spain). H23, Hop62, H157, H226 and H661 cell lines were kindly provide by Dr. R. Pio (Department of Biochemistry, School of Sciences. University of Navarra, Pamplona, Spain) [24]. HN 12, 19, 26 and 30 cell lines were kindly provided by Dr. S. Gutkind (NIDCR, NIH,MD, USA) [25].

### Western blotting and immunoprecipitation procedures

Cells were treated and collected in lysis buffer (25 mM HEPES pH 7.5, 0.3 M NaCl, 1.5 mM MgCl_2_, 0.2 mM EDTA, 1% Triton X-100, 0.1% SDS, 0.5% deoxycholic acid, 20 mM B-glycerophosphate) plus protease and phosphatase inhibitors (2 µg/ml leupeptin, 2 µg/ml aprotinin, 1 mM PMSF and 0.1 mM Na_3_VO_4_). Then, the indicated amount was loaded onto 8–10% SDS-Page and blotted against the different antibodies. In the cases of immunoprecipitation assays, extracts were washed and then, soluble fraction was incubated with the indicated antibody (1 µg/sample for 2 hr), incubated for 45 min in the presence of protein G (gamma Bind Sepharose, Amersham) and then washed 3 times in the same lysis buffer. Antibody detection was achieved by enhanced chemiluminescence (Amersham Pharmacia, Uppsala, Sweden) by film exposure or by using. Protein quantification was performed using BCA Protein Assay Kit (Pierce, Rockford, IL) following manufacturer instruction. Tubulin or total p38 MAPK were used as an internal loading control. Quantification of western blot was estimated by densitometryc analysis of filters by using WCIF Image J. software. The values (given as arbitrary units) were normalized taking into consideration the internal loading control of each sample. Results show a representative blot out of 3 with nearly identical results.

### Treatment, chemicals and antibodies

Antibodies against activated forms of p38 MAPK and MKK3/6 as well as total MKK6 were from Cell Signaling Technologies (Beverly, MA). Antibodies against total p38 MAPK and MKK3 were from Santa Cruz Biotechnology. UV irradiation was performed at 120 mJ, using a UV-Stratalinker 1800 (Stratagene) and collected 30 min after UV exposure. cDDP was purchased from Ferre Farma (Barcelona, Spain) and prepared freshly before use.

### RNA interference assays

Plasmid for lentivirus (PLKO) containing shRNA for MKK3 and MKK6 were from Sigma Aldrich (catalog number NM_002756 and NM_002578 respectively). Lentivirus production, by using 293T cells, and infections were performed as previously described [26]. 48 h after infection cells were exposed to puromycin (1,75 µg/ml for H157 and 1,25 µg/ml for H460) during 3 days for the generation of stable shRNA-expressing cell lines We selected the best performing shRNA for further analysis as judged for functional interference with endogenous levels of the target proteins by Western blotting. siRNA against MKK3 was purchased from Thermo Scientific-Dharmacon (ON-TARGETplus SMART pool L-003509-00-0005). Cells were transfected and 60 hr later were tested for endogenous expression of MKK3 and MKK6 by qRT-PCR.

### Viability assays

Viability was evaluated by the crystal violet method [12]. Cells were seeded 24-hr prior drug treatment at 4×10^4^ cells/well. Colorant was recovered using 1% acetic acid and optical density was evaluated at 590 nm. Values were referred to untreated controls. Data are the average of at least 3 independent experiments performed in triplicates cultures.

### RNA isolation, reverse transcription and real-time quantitative PCR

Total RNA was obtained with the Qiagen RNA isolation kit, following the manufacturer protocol. The isolated RNA was subsequently treated with DNase (Promega, Madison, WI) to remove any contaminating genomic DNA. The integrity of RNA was always checked by running an aliquot in an agarose gel. Reverse transcription was performed using 1 µg of DNase-treated RNA in 20 µl of reaction volume (Fermentas, Glen Burnie, MD). Samples were stored at 20°C until their utilization. Changes in the mRNA expression of MKK6/MKK3 were examined by real-time quantitative PCR using an ABIPrism 7000 Sequence Detection System (Applied Biosystems, Foster City, CA). cDNA (5 µl of diluted reverse transcription product) was amplified using SYBR1 Green PCRMaster Mix (Applied Biosystems) in the presence of primer oligonucleotides specific for MKK6, MKK3 and GADPH. The PCR conditions were as follows: 95°C for 10 min, followed by 40 cycles consisting of 95°C for 15 sec and 60°C for 1 min. The quantification was performed by the comparative cycle threshold method, using the GADPH RNA expression level as internal control. Primers for all target sequences were designed using the computer Primer Express software program specially provided with the 7000 Sequence Detection System (Applied Biosystems, Foster City, CA). In all the cases, only one amplification product was obtained. Chosen PCR primers were as follows: MKK6 sense 5′-GGCCCCTGAAAGAATAAACCC-3′, antisense 5′-CGAAGGATGGCCAACTCAATC-3′; MKK3 sense 5′-CGGCTGCAAGCCCTACAT, antisense 5′- CTCCAGACGTCGGACTTGACA-3′; GADPH sense 5′-TCGTGGAAGGACTCATGACCA-3′ antisense 5′-CAGTCTTCTGGGTGGCAGTGA-3′.

### Data analysis

Results are represented as mean ± SD of at least three independent experiments performed in triplicate. Statistical analysis was performed using the GraphPad Prism 5.00 software. Significance was determined using a t-test. The statistical significance of differences was indicated in figures by asterisks as follows: *P<0.1, **P<0.05 and ***P<0.001.

## Results

### Sensitivity to cDDP correlates with p38 MAPK activation

**Figure 1:**
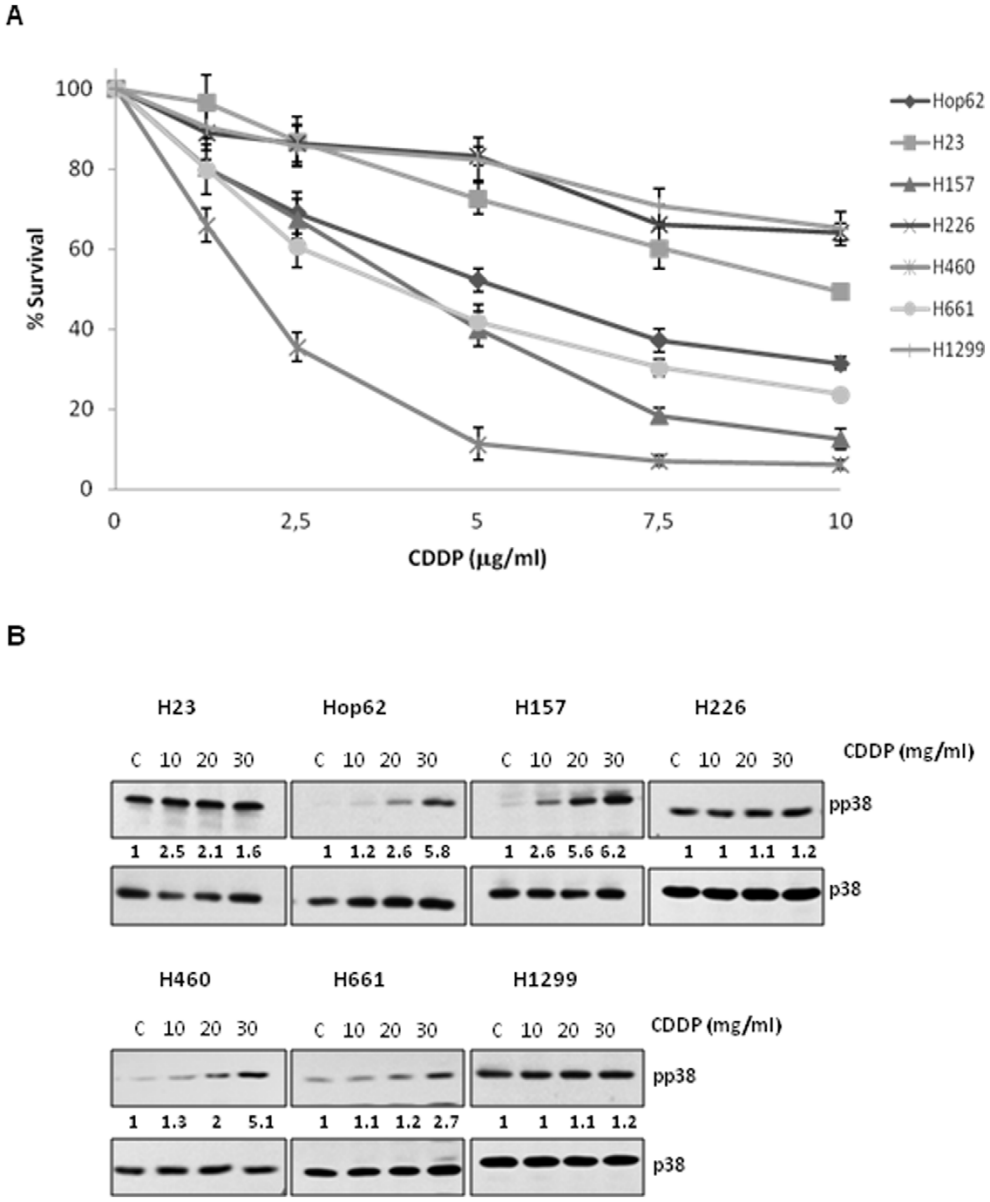
Lack of p38 MAPK activation correlates with resistance to CDDP. A) Seven NSCLC cell lines were treated with the indicated doses of cDDP during 48 hours and viability was evaluated by the crystal violet method. Survival was referred to untreated cells. Bars indicate standard deviation (SD). B) All cell lines were exposed to the indicated doses of cDDP for 2 hours. Then samples were collected and 50 µg of the total cell lysate (TCL) were used to evaluate the p38MAPK phosphorylation levels by western blotting. Membranes were re-blotted against p38 as loading control.

In order to further study the role of p38 MAPKα (referred to from now on as p38 MAPK) activation in response to cDDP, we used an experimental model comprised of 7 NSCLC (Hop62, H157, H460, H661, H226, H23 and H1299) and challenged them with cDDP. All cell lines were exposed to the indicated doses of cDDP during 24 (data not shown) or 48 hours (Fig. 1A) and viability was evaluated by crystal violet method. As it is shown, different levels of intrinsic resistance to the drug were observed. Next, phosphorylation of p38 MAPK in response to cDDP exposure was tested (Fig. 1B). Interestingly not all cell lines were able to significantly activate the kinase. While Hop62, H157, H460 and H661 were more sensitive and showed a clear activation of p38 MAPK in response to cDDP, H226, H23 and H1299 were more resistant and exhibited little response, suggesting a correlation between p38 MAPK activation capability and the response to cDDP.

### MKK6 correlates with cDDP sensitivity but is not responsible for cDDP-mediated p38 MAPK activation

**Figure 2:**
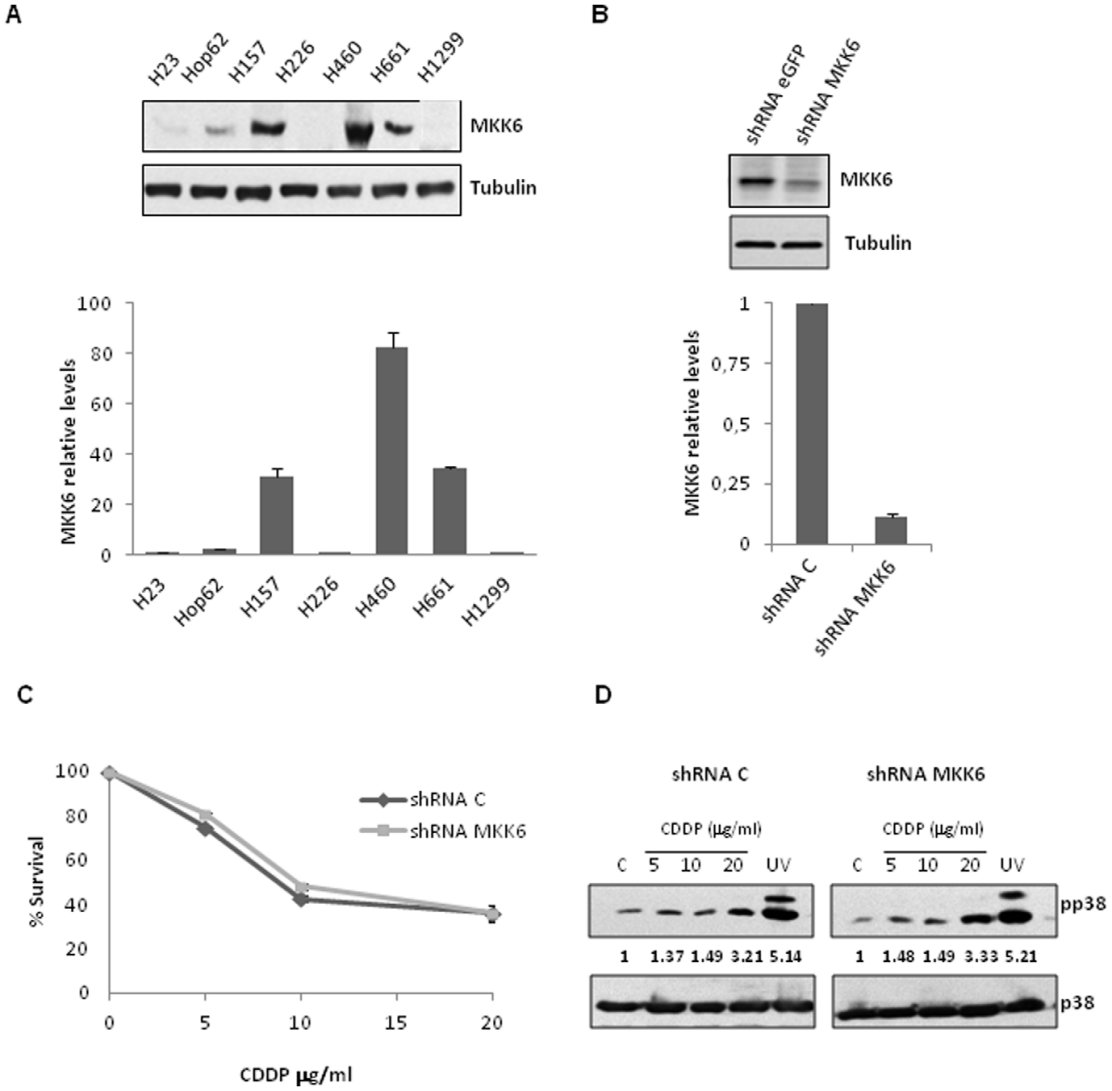
MKK6 expression level correlates with sensitivity to cDDP. A) Upper panel. Expression levels of endogenous MKK6 were evaluated in all NSCLC lines using 200 µg of the TCL. For Tubulin detection 30 µg TCL were used. Lower panel. MKK6 mRNA levels were evaluated by Q-PCR. Figure shows relative MKK6 mRNA levels referred to H23cell line. B) H157 cells were infected with lentivirus carrying an empty vector (shRNAC) or a shRNA against MKK6 (shRNAMKK6). Upper panel. 100 µg of each cell lines TCL were used to evaluate MKK6 levels by WB. Tubulin was used as loading control (30 µg TCL). Lower panel. ShRNA-induced mRNA diminution was also evaluated by Q-PCR. C) Both, shRNAC and shRNA MKK6 expressing cells were treated with the indicated doses of cDDP during 48 h. Survival was referred to untreated cells. Bars mean SD. D) H157 shRNA expressing cells were treated and analysed as in 1B. Levels of active p38 were quantified and referred to p38 total level.

The lack of p38 MAPK activation could be explained, among other possibilities, by a defect in its direct upstream activators, the MAPKKs. As one of the main activators of p38 MAPK in response to several stimuli *in vitro* and i*n vivo* [18], we decided to explore MKK6.This MAPKK was studied at the protein and RNA levels by WB and Q-PCR respectively in our NSCLC cell panel (Fig. 2A). Remarkably, we observed a clear correlation between MKK6 levels, cDDP-mediated p38 MAPK activation and sensitivity to the drug. Conversely, lower levels, almost undetectable, were observed in resistant cell lines in which cDDP was unable to induce phosphorylation of p38 MAPK. This result suggests that the lack of MKK6 expression could explain the associated chemoresistance mediated by deficient p38 MAPK activation. To investigate if MKK6 levels are responsible for cDDP response, we use an shRNA to decrease MKK6 expression in two of the cell lines with high expression. For this purpose, H157 (Fig. 2B) and H460 (Fig. S1) were infected with a lentiviral vector coding for shRNA against MKK6 or a control vector and viability in response to cDDP was then evaluated (Fig. 2C). Surprisingly, MKK6 diminution did not affect cDDP response. Moreover, p38 MAPK activation in response to cDDP was evaluated in these cells and similar activation trend was found in both control and interfered cells (Figs. 2D and S1). Thus, these set of results indicated that MKK6 is not responsible of p38 MAPK activation in response to cDDP, excluding this MAPKK in p38 MAPK-associated cDDP chemoresistance.

### Resistant cell lines display high levels of MKK3 which correlates with high basal activity of p38 MAPK

**Figure 3:**
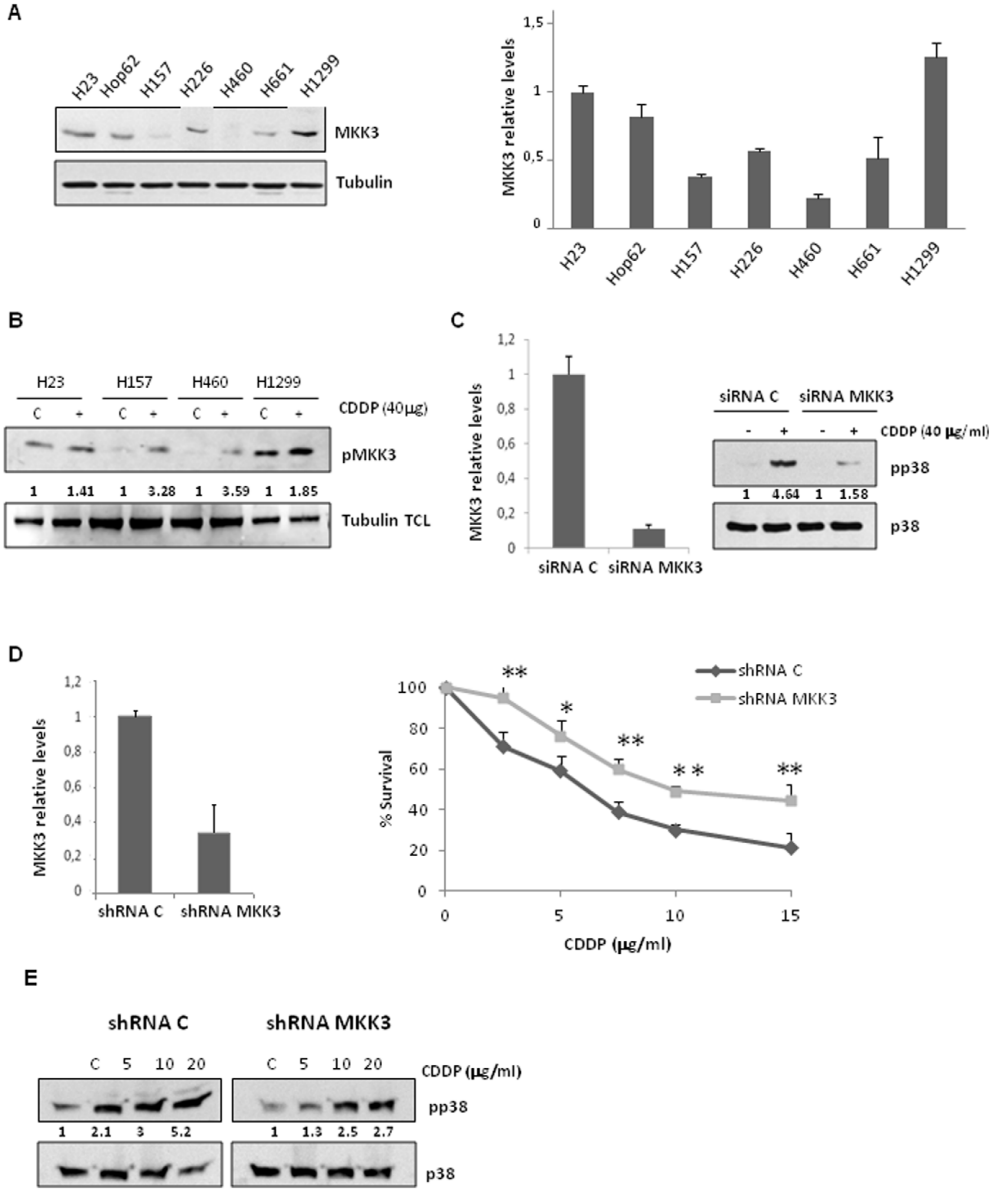
MKK3 mediates p38 MAPK dependent resistance to cDDP in NSCLC. A) MKK3 levels were analysed by WB (left panel) and Q-PCR (right panel) as in 2A. B) H23, H1299 (resistant/ high MKK3 levels), h157 and H460 (sensitive/low MKK3 levels) were exposed to cDDP (40 µg/ml). 2 h later, samples were collected and 1 mg of protein (H23, H1299) or 2 mg (H157, H460) were used to inmunoprecipitate endogenous MKK3. Inmunocomplexes were blotted against active MKK3. Equivalent amounts of TCL were used to analyze tubulin. C) H157 cells were transfected either with a siRNA C or a siRNA against MKK3 by duplicate. 60 h later mRNA MKK3 levels were evaluated by Q-PCR in a set of plates. The other set was split 1/2 and 24 hour later (left panel) cells were treated with cDDP at the indicated dose during 2 h. Cells were collected and active p38 MAPK levels were evaluated. Membranes were re-blotted against p38 as a loading control. D) H157 cells were infected with lentivirus coding for a shRNA against MKK3 or a shRNA C and stable cell lines were generated like in 2B. Left panel. MKK3 mRNA levels were evaluated by Q-PCR in control and interfered cell lines. Right panel. Stable shRNA expressing cells were processed as in 2C.(*P<0.1, **P<0.05). E) Stable shRNA expressing cells were treated and analysed as in 1B. Levels of active p38 were quantified and referred to p38 MAPK total level.

All previous evidence supports a lack of involvement of MKK6 in the response to cDDP. Therefore, we next asked if the other main activator of p38 MAPK, MKK3, could be implicated in the selective cDDP-mediated-activation of p38 MAPK and the resistance to this drug observed in our model of NSCLC derived cell lines. To investigate this possibility, MKK3 levels were evaluated in all cell lines by WB and Q-PCR (Fig. 3A), showing significant and opposite pattern to MKK6 at both protein and RNA levels (Fig. 2A). Resistant cell lines showed higher levels of MKK3 than sensitive ones but nonetheless were unable to activate p38 in response to cDDP. Taking this fact into account, we explored the capability of cDDP to induce activation of this MAPKK in both sensitive and resistant cell lines. Therefore, we challenged four of the cell lines, two sensitive (H157, H460) and two resistant (H23 and H1299), and examined MKK3 activation (Fig. 3B). Interestingly, cells with high levels of MKK3 showed an elevated phosphorylation in the absence of stimuli but then poor activation of this particular MAPKK in response to cDDP, while sensitive cell lines, with low levels, showed a more than 3 fold increase in MKK3 activation in response to cDDP. This result indicated that although sensitive cell lines express low levels of this MAPKK, cDDP can efficiently activate the MKK3-p38 MAPK signaling axis, suggesting that this MAPKK is a key player in the activation of p38 MAPK and the associated chemoresistance. To corroborate this hypothesis, we used a genetic-based approach whereby siRNA against MKK3 was used in a sensitive cell line (H157). As shown (Fig. 3C), specific knockdown of MKK3 almost completely blocked p38 MAPK activation, demonstrating the critical role of MKK3 in the p38MAPK activation mediated by cDDP. In addition, to further study the role of MKK3 in cDDP-resistance, we evaluated the response to the drug in both sensitive cell lines (H157 and H460) stably expressing an MKK3-targeted shRNA (Figs. 3D and S2). Remarkably, the diminution of MKK3 levels rendered a more resistant phenotype, increasing the survival to approximately 30% when compared to cells infected with an shRNA control (Fig. 3D), as well as a marked decrease in activation of the pathway in response to cDDP (Fig. 3E). In summary, this set of experiments supports the crucial role of MKK3 in p38 MAPK activation by cDDP and the associated chemoresistance.

### MKK3 controls MKK6 levels in NSCLC derived cell lines through activation of p38 MAPK

**Figure 4:**
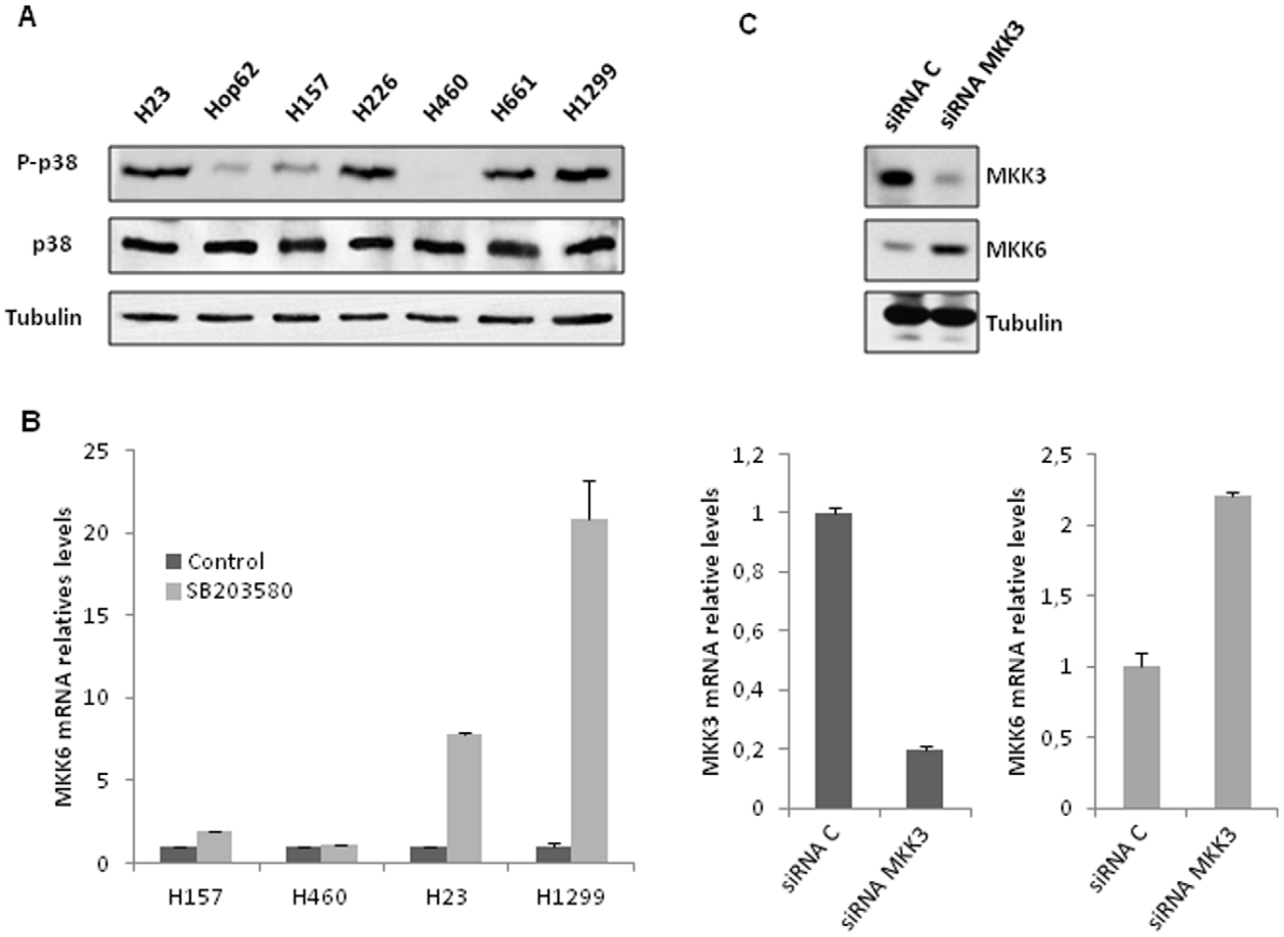
MKK3-mediated p38 MAPK activation controls MKK6 expression level. A) 50 µg of TCL was used to evaluate active p38 MAPK basal levels in the 7 NSCLC derived cell lines panel. Membrane was then re-blotted against total p38 and tubulin as loading controls. B) H157, H460 (sensitive/ high MKK6 levels), H23 and H1299 (resistant/low MKK6 levels) were exposed to 10 µM SB203580 during 36 h and MKK6 mRNA levels were evaluated by Q-PCR. The inhibitor was renovated every 12 h. C) H1299 cells were transfected with a siRNA targeting MKK3 and 72 hour later MKK3 and MKK6 levels were evaluated by WB (upper panel) using 100 ug of TCL. mRNA levels were evaluated by Q-PCR at 60 hours post transfection (lower panel).

**Figure 5:**
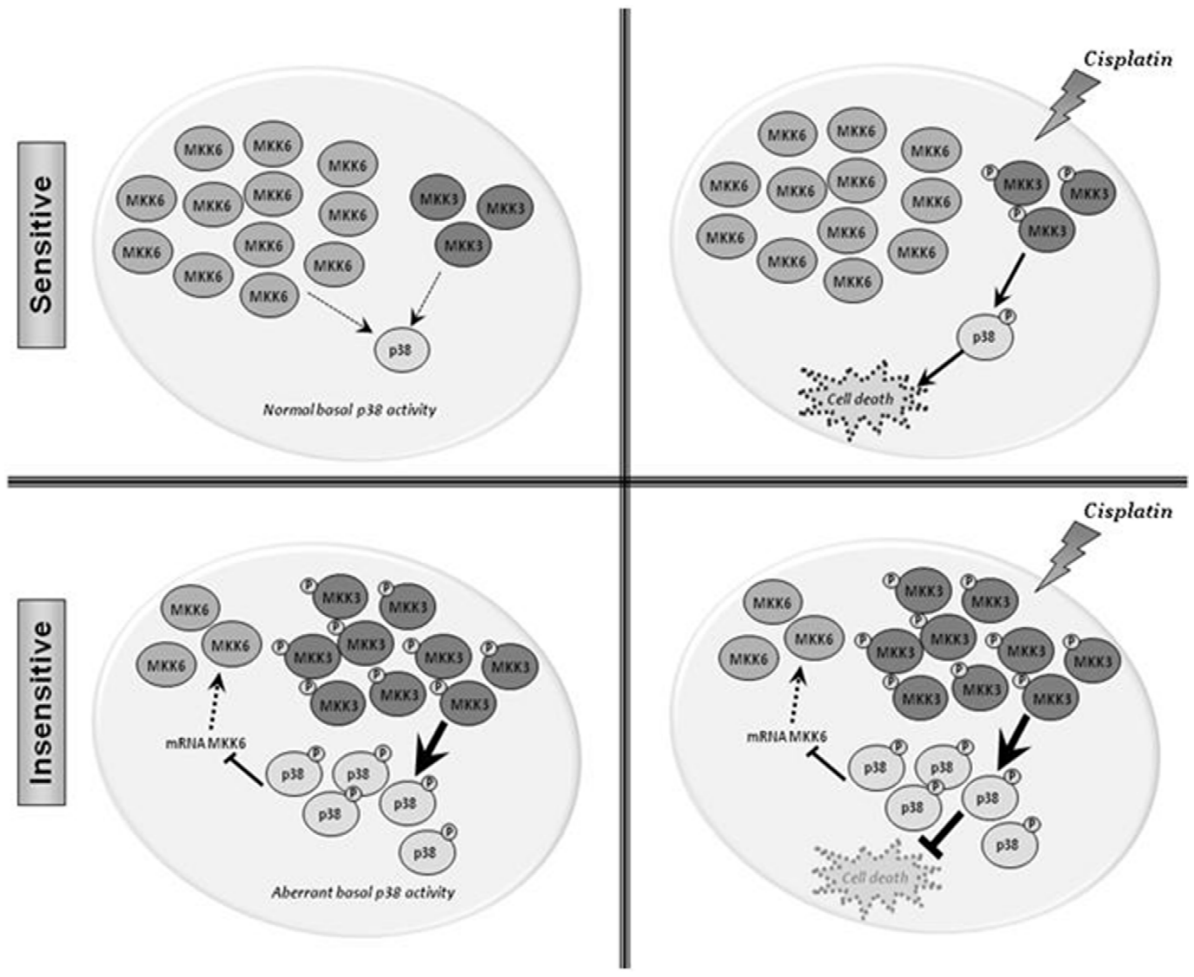
Balance between MKK6 and MKK3 mediates p38 MAPK associated resistance to cisplatin. Upper panel: In the absence of stimuli (left), sensitive cells display high basal levels of MKK6 and low basal levels of MKK3 which maintain a normal basal activity of p38 MAPK. When cells are exposed to cDDP (right), the existing molecules of MKK3 are phosphorylated and then they are able to activate p38 MAPK, provoking the death of the cells. Lower panel: In basal conditions (left), resistant cells express elevated levels of MKK3 which is indeed intrinsically phosphorylated. As a consequence, resistant cells show an aberrant basal activity of p38 MAPK which is controlling MKK6 by decreasing its RNA levels. As consequence of the constitutive activation of MKK3, cells are unable to respond to cDDP treatment (right), surviving after exposure to the drug.

In light of our previous findings which show an opposite expression pattern of both MAPKKs in our NSCLC experimental model, we decided to explore the molecular basis of differential expression for MKK6/MKK3. In this regard, p38MAPK has been previously demonstrated to be a potent regulator of MKK6 RNA levels in mouse embryonic fibroblasts (MEF) [19]. To evaluate the influence of the p38 MAPK phosphorylation in the regulation of MKK6 levels in a tumorigenic context, basal levels of active p38 MAPK were evaluated. Interestingly, we observed a marked correlation between intrinsic activation of p38 MAPK and MKK3 levels (Fig. 4A and Fig. 3A) that exhibited an inverse correlation with MKK6 (Fig. 2A). Therefore, to investigate the contribution of p38 MAPK basal activity in the regulation of MKK6 levels, we measured the RNA levels of this MAPKK in the presence of SB203580, a potent inhibitor of p38 MAPK activity [27]. As it is shown in Fig. 4B, the presence of SB203580 increased MKK6 RNA levels in resistant cells (H23 and H1299) cells which possess high basal levels of phosphorylated p38 MAPK, while barely affecting its levels in sensitive cells (H157 and H460) cells which have low levels of basal p38 MAPK activation. To more fully support our observation, we used an RNA interference approach. Remarkably, knockdown of MKK3 by siRNA in the resistant cell line H1299, which has high levels of MKK3, rendered an increase in MKK6 protein and RNA levels (Fig. 4C). In summary, this set of results strongly suggests that activation of p38 MAPK through MKK3 controls MKK6 levels in NSCLC (Fig. 5).

### MKK3-p38 MAPK signaling axis is also a major determinant of cellular response to cDDP in Head and Neck Squamous Cell Carcinoma derived cell lines

**Figure 6:**
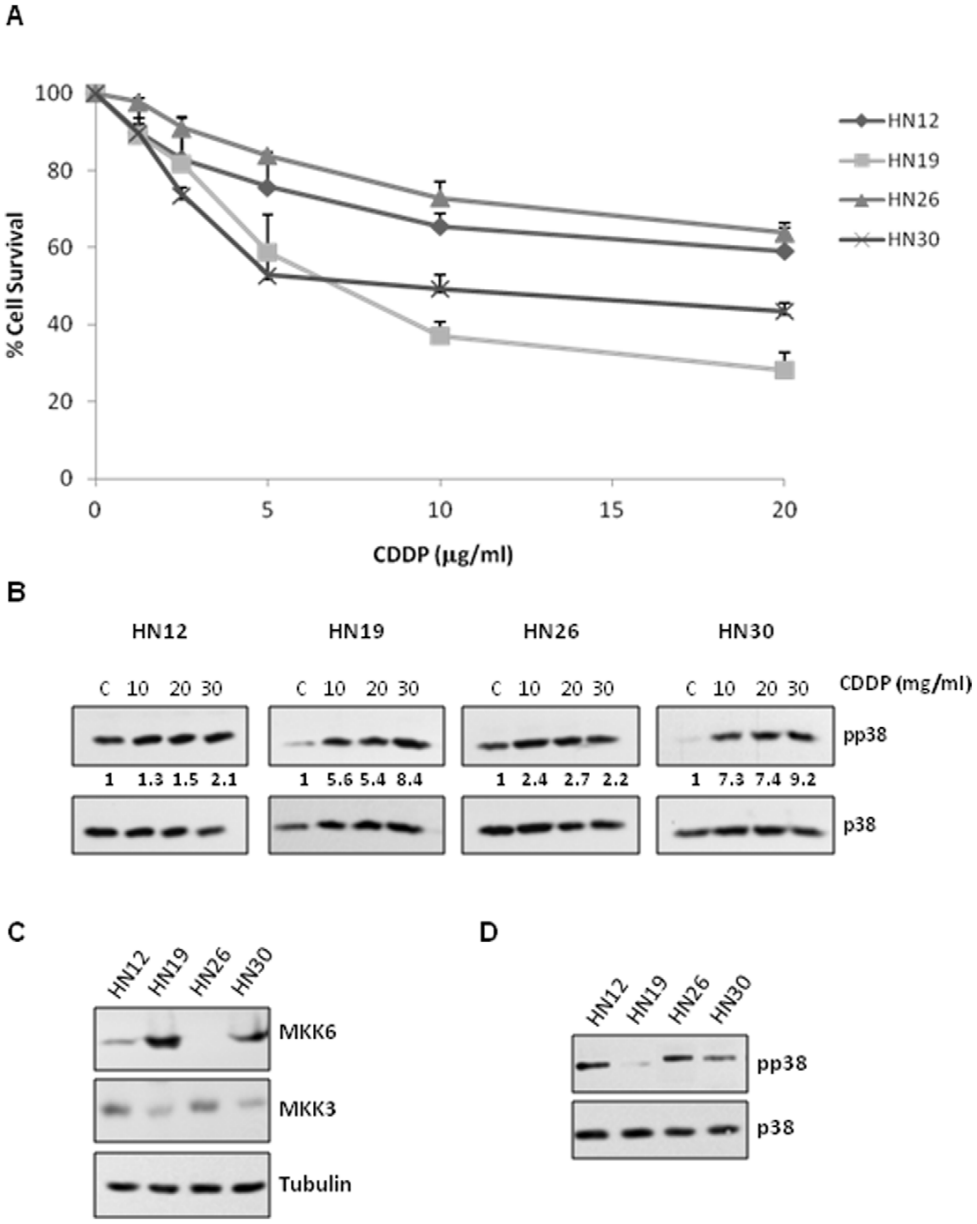
Basal p38 MAPK activation and MKK3 levels correlates with cDDP resistance in HNSCC derived cell lines. A) 4 HNSCC derived cell lines were treated with the indicated doses of cDDP during 48 hours. Survival was referred to untreated cells. Bars indicate standard deviation (SD). B) All cell lines were exposed to the indicated doses of cDDP for 2 h. Then, samples were collected and 50 µg of the total cell lysate TCL were used to evaluate the p38MAPK phosphorylation levels. Membranes were re-blotted against p38 as loading control. C) 100 µg of TCL was used to evaluate MKK6 and MKK3 protein levels. Tubulin was used as loading control. D) 50 µg of TCL were use to estimate levels of basal phosphorylation p38 MAPK and total p38 MAPK expression levels.

We have demonstrated that MKK3 is a key player in cDDP-mediated p38 MAPK activation and its associated cellular resistance in NSCLC. However, we aimed to examine whether our model was applicable to other pathologies such as head and neck squamous cell carcinoma (HNSCC), where cDDP is also a critical therapeutic agent [28]. To this end, a panel of 4 cell lines derived from different oral tumors was used (HN12, HN19, HN26 and HN30). First, we evaluated viability in response to cDDP and found varying sensibility to the drug, in a similar way to NSCLC (Fig. 6A). Next, we studied the capacity of cDDP to activate the p38 pathway in these HNSCC derived cell lines. For this purpose, cells were exposed to different doses of cDDP and activation of p38 was evaluated (Fig. 6B). While HN19 and HN30 were more sensitive and showed a marked activation after drug exposure, HN12 and HN26 were more resistant and exhibited only moderate activation. Next, we evaluated MKK3 and MKK6 levels in our HNSCC cell line panel. Remarkably, as observed for NSCLC derived cell lines, resistant cell lines (HN12 and HN26) exhibit low levels of MKK6 and high levels of MKK3, while sensitive cell lines (HN19 and HN30) display high levels of MKK6 and low levels of MKK3 (Fig 6C). Hence, to complete this set of experiments, we evaluated intrinsic p38 MAPK activation levels. As expected, resistant cell lines (HN12 and HN26) exhibited higher basal phosphorylation levels than intermediate and sensitive cell lines (HN30 and HN19, respectively) (Fig. 6D). In summary, all this data support our previous results in NSCLC, suggesting the potential use of MKK6/MKK3 as biomarker in cisplatin based therapy and confirming our proposed model also for HNSCC (Fig. 5).

## Discussion

In this report we reveal the critical role of the p38 MAPK network in the response to cDDP in NSCLC cell lines. Moreover, we provide mechanistic insights into the resistance to cDDP through the altered balance between MKK3/MKK6, and propose the use of these MAPKK as new biomarkers.

With regards to the p38 MAPK signaling pathway, our data demonstrates a unique intrinsic control that has not been described for other MAPK signaling pathways. In its regulation, one of its main MAPKKs (MKK3) is able to control another (MKK6) through the activation of the p38 MAPK, supporting previous observations [19]. It is interesting to note that our data are obtained from transformed cell lines while the previous observations were made in the context of non-transformed cells (MEF) from the KO mice for p38 MAPK, suggesting that our proposed mechanism is one of the natural ways by which the p38 MAPK signaling pathway can be deregulated in cancer. In this regard, p38 MAPK has been proposed to act as a tumor suppressor by sensing ROS levels [29]. In fact, the lack of p38 MAPK phosphorylation correlates with an increase in tumorigenesis in several different contexts [18], [21], [29] suggesting that the activation of the pathway should be opposite to transformation (for a review [20], [30]). However, other evidence supports a role for p38 MAPK in survival and proliferation in both normal and transformed cells (for revision see [31]). In our experimental model we found a significant disparity in the basal levels of active p38 MAPK, meaning that constitutive activation of the p38 MAPK is not a universal phenomena in cancer, or at least NSCLC. Whether this differing status of p38 MAPK, due to an imbalance between MKK6/MKK3, might be implicated in the genesis of NSCLC or in other types of tumors, as well as other aspects of tumor biology (eg. metastasis, invasiveness), needs to be further studied.

Our data reinforce the idea that p38 MAPK is a major player in cDDP resistance [12], [32]. In addition, our report introduces a new concept concerning the role of p38 MAPK in cDDP resistance. Here, we demonstrate that the inability of cDDP to activate p38 MAPK correlates with chemoresistance and this resistance is a consequence of basal hyperactivation of the pathway. Interestingly, ovarian cell lines with acquired resistant to cDDP, generated by co-culture with the drug, showed elevated basal activation levels of p38 MAPK [15], as well as in the case of resistant human keratinocytes obtained with the same protocol (Galan-Moya and Sanchez Prieto, unpublished observations). In light of our finding, we can state that constitutive activation of the pathway seems to be a natural mechanism of resistance associated with p38 MAPK. This possibility is particularly important considering that no genetic/epigenetic mechanisms (eg, mutations, variation in gene copy number, LOH, methylation of promoters, etc.) has been described so far for p38 MAPK. It's noteworthy that several screenings in yeast and eukaryotic cells have proposed key molecules in the DNA damage response –vg. BRAC1, BRCA2 or ATR- as critical mediators in cDDP response [33] and, indeed, some of them has been connected with the p38 MAPK signaling pathway in response to several antitumor agents, including cDDP, doxorubicin and 5-Fluorouracil [34], [35]. Nonetheless, other molecules implicated in this signaling pathway, such as ATF3 [36] and MKP1, which has been related to both “de novo” and acquired resistance to cDDP in ovarian and lung cancer models [17], [37] should be considered in the final role of p38 MAPK in cDDP resistance.

Another remarkable conclusion from this report is the differential roles of MKK3 and MKK6 in the control of the p38 MAPK signaling pathway in response to cDDP. Of note, several reports support the existence of a connection between p38 MAPK and MKK6 in the response to cDDP [12], as well as in response to others DNA damaging agents, such as ionizing radiation [15], [38]. However, it has been also shown how inhibition of ASK1, a MAPKKK of the p38 MAPK signaling pathway, affect MKK3 in the apoptotic response to cDDP [39]. In the present work, we demonstrate that the activation of p38 MAPK in response to cDDP appears to be connected to the basal expression/activity of MKK3, which controls basal levels of active p38 MAPK, explaining why overactivation of both MAPKK is not possible, since one regulates the levels of the other. The specific role of MKK3 in the response to cDDP in NSCLC model is not unique. Indeed , several reports have already shown that certain stimuli can activate biological response depending exclusively on MKK3, as in the case of selenite [40], hyperglycaemia induced by low doses of streptozotocin [41] or in the case of TGF-β1 [42], [43]. Furthermore, in some cases it has been proven that the activation of p38 MAPK though MKK3 is implicated in the biological properties of tumors, as is the case for gliomas or HNSCC [44], [45]. Thus, in spite of apparent redundancies between both MAPKK reported in some cases, our data support that there are some biological processes in which they display differential roles, as we have demonstrated here for cDDP resistance in NSCLC derived cell lines.

Finally our work proposes that the balance between MKK6/MKK3 could be used as a reporter for p38 MAPK activity and, therefore, as a putative new biomarker in cancer, particularly in NSCLC. In addition to being a major intermediary in the response to cDDP, p38 MAPK is also implicated in some of the side effects provoked by cDDP such as nephrotoxicity [46]. Consequently, evaluation of the status of this signaling pathway is of interest for determining the likelihood of both chemoresistance and cDDP-associated side-effects. In this regard, there is little evidence of the potential role of this signaling pathway as a biomarker in either lung or head and neck cancers [22], [23], [45]. As we have demonstrated here, the chemoresistance observed in both NSCLC and HNSCC is due to a lack of activation of the pathway in response to cDDP, indicating that the ideal situation to validate p38 MAPK signaling pathway as a biomarker would be collecting fresh samples before and after treatment, which together with technical challenges (biopsies must be properly collected to preserve phosphorylation) reduce the feasibility of measuring phosphorylated p38 MAPK levels as a biomarker. However, our data indicates that constitutive hyperactivation of the pathway mediates chemoresistance and the expression levels of MKK6/MKK3 are a suitable readout of p38 MAPK activity. Therefore, evaluation of both MAPKK could be a way to estimate chemoresistance without the necessity to evaluate phosphorylation, thereby simplifying the detection of the p38 MAPK status. Hence both MAPKK (MKK6/MKK3) could be included in the growing list of markers aimed to predict the outcome to cDDP, such as ERCC1 or BRAC1 (for a review [47]). In addition to this, new therapies based on antibodies- or pharmacologically-mediated inhibition of EGFR have been proposed for NSCLC [48]. Now, several evidences support a decisive role for p38 MAPK signaling pathways in the biology of EGF receptor [16], [49]–[51]. Therefore , our observation about p38 MAPK signaling pathway in the cellular response to cDDP, could be also be involved in these novel and selective therapies, that could account for the future personalized therapy based on EGFR status as well as downstream molecules as K-Ras [52]. Furthermore, p38 MAPK could be a novel convergence point in NSCLC, considering that cDDP and EGRF based therapies seem to be interrelated [53], [54].

In summary, our data provides new insights into the task of the p38 MAPK pathway in cDDP resistance. This effect is mediated through constitutive activation of the pathway in which MKK3 appears to be the key protein, while MKK6-dependent signaling is almost marginal. Whether these observations could be applicable to other pathologies is currently being studied, as well as putative proteins that potentially regulate the MKK3-p38 MAPK axis.

## Supporting Information

Figure S1 **Effect of MKK6 interference in H460 cells.** H460 cells were infected with lentivirus carrying an empty vector (shRNAC) or a shRNA against MKK6 (shRNAMKK6). A) ShRNA-induced mRNA diminution was evaluated by Q-PCR as in H157 cell line. B) shRNAC and shRNA MKK6 expressing cells were treated with the indicated doses of cDDP during 48 h. Survival was referred to untreated cells. Bars mean SD. C) shRNA expressing cells were treated and analysed as in Figure 1B. Levels of active p38 MAPK were quantified and referred to p38 MAPK total level.
(TIF)

Figure S2 **Effect of MKK3 interference in H460 cells.** H460 cells were infected with lentivirus carrying an empty vector (shRNAC) or a shRNA against MKK3 (shRNAMKK3). A) shRNAC and shRNA MKK3 expressing cells were treated with the indicated doses of cDDP during 48 h. Survival was referred to untreated cells. Bars mean SD. B) ShRNA-induced mRNA diminution was evaluated by Q-PCR as in H157 cell line. C) shRNA expressing cells were treated and analysed as in Figure 1B. Levels of active p38 MAPK were quantified and referred to p38 MAPK total level.
(TIF)
