# Supplementary figures and images for: Balance between MKK6 and MKK3 Mediates p38 MAPK Associated Resistance to Cisplatin in NSCLC
Source: PLoS One. 2011 Dec 2;6(12):e28406. doi: 10.1371/journal.pone.0028406 (PMC3229586; doi:10.1371/journal.pone.0028406)

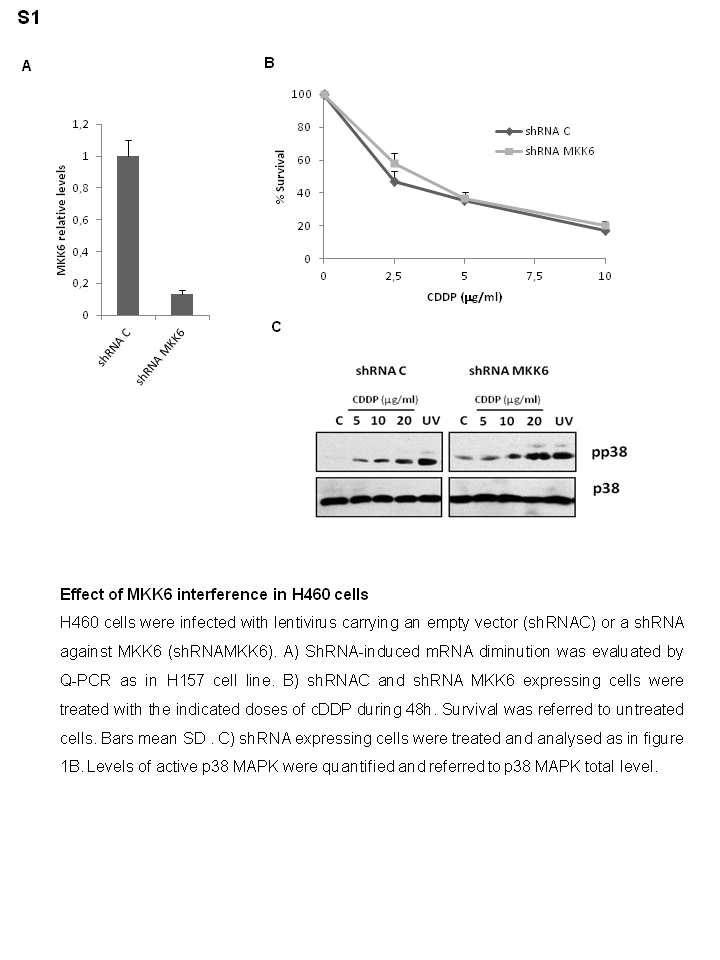

Supplement: Figure S1 — Effect of MKK6 interference in H460 cells. H460 cells were infected with lentivirus carrying an empty vector (shRNAC) or a shRNA against MKK6 (shRNAMKK6). A) ShRNA-induced mRNA diminution was evaluated by Q-PCR as in H157 cell line. B) shRNAC and shRNA MKK6 expressing cells were treated with the indicated doses of cDDP during 48 h. Survival was referred to untreated cells. Bars mean SD. C) shRNA expressing cells were treated and analysed as in Figure 1B. Levels of active p38 MAPK were quantified and referred to p38 MAPK total level. (TIF) [file pone.0028406.s001.tif]

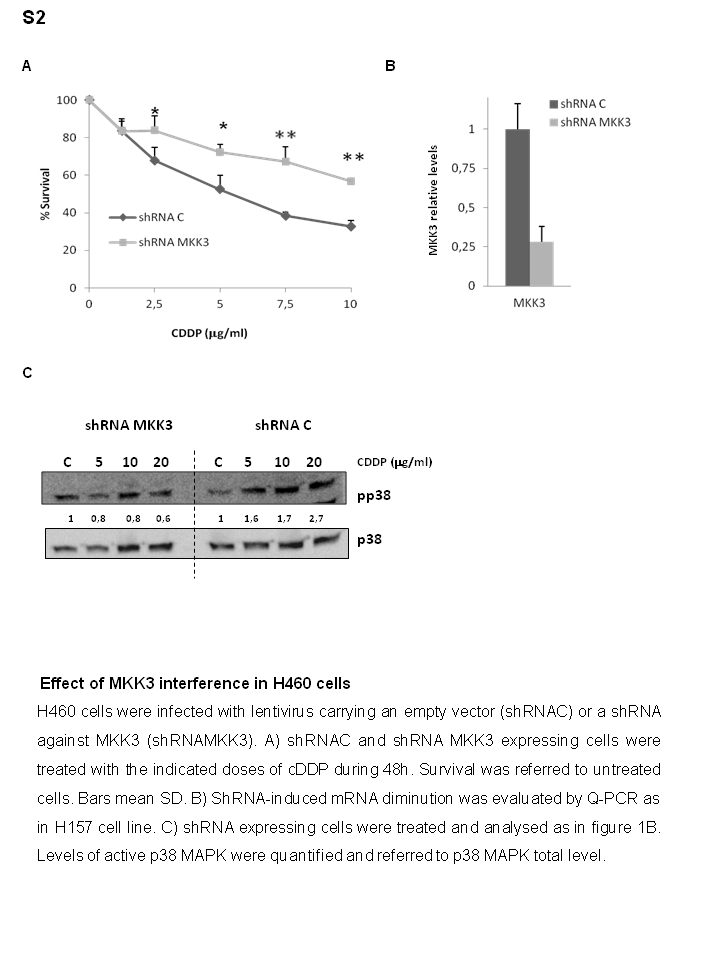

Supplement: Figure S2 — Effect of MKK3 interference in H460 cells. H460 cells were infected with lentivirus carrying an empty vector (shRNAC) or a shRNA against MKK3 (shRNAMKK3). A) shRNAC and shRNA MKK3 expressing cells were treated with the indicated doses of cDDP during 48 h. Survival was referred to untreated cells. Bars mean SD. B) ShRNA-induced mRNA diminution was evaluated by Q-PCR as in H157 cell line. C) shRNA expressing cells were treated and analysed as in Figure 1B. Levels of active p38 MAPK were quantified and referred to p38 MAPK total level. (TIF) [file pone.0028406.s002.tif]
